# Rosoideae-specific duplication and functional diversification of *FT-like* genes in Rosaceae

**DOI:** 10.1093/hr/uhac059

**Published:** 2022-03-08

**Authors:** Xiao-Dong Jiang, Mi-Cai Zhong, Xue Dong, Shu-Bin Li, Jin-Yong Hu

**Affiliations:** 1 CAS Key Laboratory for Plant Diversity and Biogeography of East Asia, Kunming Institute of Botany, Chinese Academy of Sciences, 650201 Kunming, Yunnan, China; 2 Germplasm Bank of Wild Species, Kunming Institute of Botany, Chinese Academy of Sciences, 650201 Kunming, Yunnan, China; 3 Flower Research Institute, Yunnan Agricultural Academy of Sciences, Kunming 650231, China; 4 Kunming College of Life Science, University of Chinese Academy of Sciences, 650204 Kunming, Yunnan, China

Dear Editor,

Rosaceae plants provide some of the most important fruits and flowers, like apple, peach, pear, strawberry, and rose. Understanding the molecular genetic mechanisms that underlie the regulation of flowering time, i.e. the transition from vegetative to reproductive growth, is therefore essential for securing flower and fruit productivity. At least five pathways that regulate flowering in model plants have been well characterized. Although components in the flowering pathways may differ among species, most endogenous and exogenous cues are integrated into several key and conserved hubs, including the florigen that is encoded by *FLOWERING LOCUS T* (*FT*). Expressed in vasculature cells and transported to shoot apical meristems, FT interacts with the bZIP transcription factor FD and a scaffold protein 14-3-3 to form a florigen protein complex that induces the expression of inflorescence and floral meristem genes. Because of its pivotal roles in flowering time control and other developmental processes, the regulation of *FT* expression occurs at the transcriptional, post-transcriptional, and translational levels [1]. Gene copy-number variation via random/tandem duplication or whole genome duplication (WGD) accompanied by functional diversification provides another regulatory layer for *FT* function, and duplication of *FT-like* genes correlates tightly with crop domestication in rice, maize, and soybean [2–4]. However, this has not been investigated systematically in Rosaceae.

**Figure 1:**
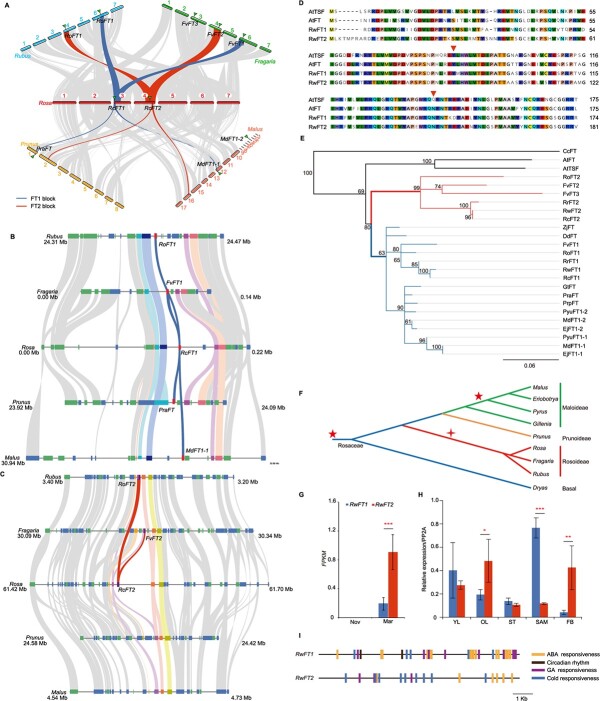
Lineage-specific random duplication of *FT-like* genes followed by functional diversification in Rosaceae. A. Macro-synteny comparisons between *Rosa chinensis* ‘Old Blush’ (OB) and four Rosaceae species (*Ro, Rubus occidentalis*; *Fv, Fragaria vesca*; *Pra, Prunus armeniaca*; *Md, Malus domestica*). Lines show the syntenic regions among genomes. Collinear regions surrounding *FT-like* genes are colored in blue and red lines for *FT1* and *FT2*, respectively. Triangles mark the positions of *FT* genes in the syntenic blocks. B. Micro-synteny relationships for *FT1* blocks among the four Rosaceae species relative to OB. The blue lines mark the *FT1* gene. C. Micro-synteny relationships for *FT2* blocks among the four Rosaceae species relative to OB. The red lines mark the *FT2* gene. Note that no signs of *FT2-like* genes were present in *Prunus* and *Malus*, despite the good collinearity of the surrounding regions in the five genomes. D. An alignment of the FT-like proteins in *Arabidopsis* and rose (BT). The downward red triangles mark the two amino acids that distinguish the FT-like and TFL1-like proteins. E. A phylogenetic clustering of FT-like proteins in Rosaceae, jujuba, and *Coptis* plants, with *Arabidopsis* PEBPs as outgroups (removed from this tree). *Arabidopsis* FT and TSF were also included. Red and blue branches mark the FT2-like and FT1-like groups in Rosaceae plants. Numbers on branches indicate the support percentages based on 1000 bootstrap replicates. *Rw*, *Rosa wichuraiana*; *Rc*, *R. chinensis*; *Rr*, *Rosa rugosa*; *Md*, *M. domestica*; *Pyu*, *Pyrus ussuriensis* × *communis*; *Fv*, *F. vesca*; *Ro*, *R. occidentalis*; *Ej*, *Eriobotrya japonica*; *Gt*, *Gillenia trifoliata*; *Pra*, *P. armeniaca*; *Prp*, *Prunus persica*; *Dd*, *Dryas drummondii*; *Zj*, *Ziziphus jujuba*; *Cc*, *Coptis chinensis*. F. A simplified model showing the Rosoideae-specific duplication of *FT2-like* genes (marked by a red cross) in Rosaceae. The other two subfamilies of Rosaceae (Prunoideae and Maloideae) did not feature such a duplication. Red stars indicate the WGD event shared by all Rosaceae plants and the WGD specific to Maloideae plants. G. Differential expression of *FT-like* genes in BT leaves collected in March (reproductive phase) and November (vegetative phase). The Y-axis shows the mean FPKM values for both genes with standard deviation. H. Tissue-specific differentiation of *FT-like* gene expression in BT. The expression was examined in three biological replicates via quantitative RT-PCR using rose *PP2A* as the reference gene. YL, young leaves still closed; OL, leaves just opened; ST, young stem 1 cm below the shoot apical tip; SAM, shoot apical meristem tissues without leaves; FB, flower buds prior to anthesis. I. Distribution of conserved *cis*-motifs related to ABA (orange), the circadian clock (brown), GA (purple), and low temperature responses (blue) along the 10-kb promoter regions of the two *FT-like* genes in BT.

Here, we report the duplication and expression diversification of *FT* in rose, an emerging woody model for flowering regulation. We identified two copies of *FT* via BLAST in the genomes of *R. wichuraiana* ‘Basye’s Thornless’ (BT; *RwFT1*, *Rw3g00010*; and *RwFT2*, *Rw0G011750*) [5] and *Rosa chinensis* ‘Old Blush’ (OB; *RcFT1*, *RchiOBHm_Chr3g0447431*; and *RcFT2*, *RchiOBHm_Chr4g0439111*) [6] (Fig. 1). An inter- and intra-genome macro-synteny analysis with MCScanX in five Rosaceae plants with chromosome-level genome assemblies revealed that the loci harboring rose *FT1* and *FT2* shared high levels of collinearity between OB and *Rubus*, *Fragaria*, *Prunus*, and *Malus* (Fig. 1A). A further detailed gene collinearity analysis demonstrated that the rose *FT1* locus was syntenic to the *FT1* loci of the other four Rosaceae plants (Fig. 1B). Despite the fact that the four genes up- and downstream of *FT2* were highly collinear in all five Rosaceae species, no orthologous *FT2* was detected in *Prunus* and *Malus* (Fig. 1C). Rose *FT2* was also not listed in the syntenic gene pairs generated via the WGD event in Rosaceae [5]. A subsequent BLAST scan with reduced criteria in the genomes of these two species detected no sign of *FT2*-*like* sequences, implying that *FT2-like* genes may have arisen from random duplication in Rosoideae or may have been lost in the common ancestor that gave rise to the Maloideae and Prunoideae lineages.

Comparison of rose FT1 and FT2 protein sequences with Arabidopsis FT and TSF demonstrated that rose FT2 differed from FT1 at 29 positions, with FT2 being seven amino acids longer than FT1. The two amino acids that distinguish FT (Tyr85 and Gln140) from TFL1 were conserved in both FT1 and FT2 (Fig. 1D). Detailed protein structure analyses demonstrated that both FT proteins contained the highly conserved PEBP domain. A further phylogeny reconstruction with FT-like proteins from twelve Rosaceae and two outgroup species (*Ziziphus jujuba*, Rhamnaceae, and *Coptis chinensis*, Ranunculaceae) identified two major clades (Fig. 1E). Clade I contained *FT1-like* genes from all 12 Rosaceae species and jujube, whereas clade II contained *FT2-like* genes from only Rosoideae (*Rosa*, *Fragaria*, and *Rubus*). Consistent with the recent WGD in Maloideae, *Malus*, *Eriobotrya*, and *Pyrus* have two *FT1-like* genes (Figure 1E) [5]. Because no recent WGD event is present in the Rosoideae, these data indicate that a Rosoideae-specific duplication of *FT2-like* genes may have occurred prior to the separation of all Rosoideae plants from their common ancestor but after the separation of Rosoideae from Rosaceae (Fig. 1F). Interestingly, these *FT2*s were grouped together with the known rose *FT* and strawberry *FvFT2* and *FvFT3,* which act as flowering promoters [4, 7]. However, the roles of rose *FT1-like* genes have never been investigated.

Therefore, we next compared the expression of the two *FT*s using transcriptome data from BT leaves harvested in November (non-flowering season) and March (flowering season) [8]. No reads were identified for either gene in leaves harvested in non-flowering season, whereas a significant difference in expression was observed in leaves harvested during the flowering season: *RwFT2* expression was four times higher than that of *RwFT1* (Fig. 1G). With a RT-qPCR approach, we next compared their expression in five tissues of BT, for which the flowering had started (Fig. 1H). *RwFT1* expression was about six-fold higher than that of *RwFT2* in shoot apical tissues. On the other hand, *RwFT2* expression was significantly higher than *RwFT1* expression in both open leaves and flower buds prior to anthesis. Both genes were expressed at similar levels in young leaves and young stems about 1 cm below the shoot apical tissues. These findings indicate that the two *FT*s have diverged in their expression. Consistent with this finding, a detailed examination of the 10 kb upstream of the translation initiation site showed significant variation in the numbers of potential *cis-*elements related to hormones (ABA and GA), circadian rhythm, and cold responses (Fig. 1I). In line with its relatively high expression in shoot apical tissues, the promoter of *RwFT1* contained fifteen and eight cis-elements related to ABA and GA responses, whereas the *RwFT2* promoter featured only six and three, respectively. The *RwFT1* promoter harbored two *cis-*elements related to circadian rhythm regulation, whereas *RwFT2* featured none. Conversely, the *RwFT2* promoter had more *cis-*motifs related to cold temperature (nine) than *RwFT1* (five).

In summary, we identified a new and Rosoideae-specific *FT* paralog generated from random duplication. As in strawberry, *FT1* had diverged in sequence and expression pattern from the well-known *FT2-like* in rose [7]. Given the essential roles played by hormones in bud dormancy and flowering time regulation in woody perennials [9, 10], it is likely that rose *FT1* serves as an important hub, integrating signals from hormones and the circadian clock as well as branching. Rose *FT2* may play more roles in the response to cold stimulus. It is also possible that *FT2* may regulate flower bud development or flower anthesis, possibilities that clearly await further investigation with additional molecular and genetic approaches.

## Data Availability

Relevant data can be found within the paper and are available from the corresponding authors upon request.
